# Interactions of different drug resistance mutations in HIV Reverse Transcriptase in defining patterns of drug resistance

**DOI:** 10.1186/1742-4690-8-S2-O14

**Published:** 2011-10-03

**Authors:** Mark A Wainberg

**Affiliations:** 1 McGill University AIDS Centre, Lady Davis Institute, Jewish General Hospital, Montreal, Quebec, Canada

## Background

The M184V mutation in HIV RT confers high-level resistance to both 3TC and FTC and also has a strong negative impact on viral replication capacity. In addition, the presence of M184V is usually preceded by a M1841 mutation that results from a G→A hypermutation. However, viruses that contain M184V usually outcompete M184I due to higher replication ability; therefore M184V becomes predominant. In contrast, the E138K mutation in HIV-1 RT confers resistance to Rilpivirine (RPV) and Etravirine(ETR), two NNRTIs, and also impairs viral replication capacity. Phase III clinical trials (ECHO & THRIVE ) showed that E138K/M184I are the most frequent mutations in patients who received RPV together with co-formulated TDF/ FTC,suggesting that E138K might be a signature mutation for both ETR and RPV. We have assessed the impact of E138K on enzymatic processivity, viral replication capacity, and drug susceptibility in the context of M184I/V.

## Methods

We studied recombinant HIV-1 RT enzymes and viruses containing either the E138K and M184I/V mutations alone and E1 38K-M1 84I/V in combination in regard to enzyme processivity, polymerization rates, replication capacity, and drug susceptibility.

## Results

Tissue culture selections with ETR yielded E138K that conferred modest (3-5-fold) resistance to this drug as well as to efavirenz(EFV) and neviripine (NVP). These susceptibilities were not altered in either viruses or RT enzymes containing E138K together with M184I or M184V. The relative replication capacity of HIV-1 containing either E138K or M184I/V alone was decreased by ~2- fold compared to wild-type (wt). In contrast, the simultaneous presence of E138K and M184I/V was shown to reverse this low fitness, and this result was confirmed in cell-free experiments in which purified RT enzymes containing E138K alone or E138K-M184I/V double mutations displayed higher processivity than eitherwt or M184I/V RT, especially at low dNTP concentrations. No significant differences were observed between M184I and M184V in regard to these characteristics. In addition, RT containing E138K or E138K together with M184I/M184V possessed lower RNase H activity than either wt RT or M184I/V RT.

## Conclusions

The M184I/V mutations are the most prevalent among treated individuals, but are only rarely found in transmitted resistance due to their adverse impact on viral replicative f tness. E138K has the potential to be an important signature mutation for both ETR and RPV. E138K can alsorestore the enzymatic processivity and viral replication capacity of HIV-1 variants harboringM184I/V. If E138K is able to function as a compensatory mutation for M184I/V, this may have clinical and public health importance in regard to treatment failures involving ETR, RPV, and other novel NNRTIs as well as on the detectability of these mutations in transmitted resistance. These are the first reports of compensatory mutations for the M184I/V mutations in HIV-1 RT.

